# Targeting of glutamine transporter ASCT2 and glutamine synthetase suppresses gastric cancer cell growth

**DOI:** 10.1007/s00432-018-2605-9

**Published:** 2018-02-12

**Authors:** Jianxin Ye, Qiang Huang, Jie Xu, Jinsheng Huang, Jinzhou Wang, Wenjing Zhong, Wannan Chen, Xinjian Lin, Xu Lin

**Affiliations:** 1 Key Laboratory of Ministry of Education for Gastrointestinal Cancer, School of Basic Medical Sciences, Fujian Medical University, 1 North Xuefu Road, Minhou, Fuzhou, 350108 Fujian China; 2 Department of Gastrointestinal Surgery, The First Affiliated Hospital of Fujian Medical University, Fuzhou, China; 3 Fujian Key Laboratory of Tumor Microbiology, Fujian Medical University, Fuzhou, China

**Keywords:** Glutamine transporter, Glutamine synthetase, Targeted therapy, Gastric cancer

## Abstract

**Purpose:**

Glutamine (Gln) is essential for the proliferation of most cancer cells, making it an appealing target for cancer therapy. However, the role of Gln in gastric cancer (GC) metabolism is unknown and Gln-targeted therapy against GC remains scarce. The aim of this study was to investigate the relevance of Gln in GC growth and targeting.

**Methods:**

Expression of Gln transporter ASCT2 and glutamine synthetase (GS) in the parental and molecularly engineered GC cells or in human GC specimens was determined by RT-PCR and western blot analysis or immunohistochemistry. Cell proliferation and survival was assessed by CCK-8 assay and colony formation assay. Intracellular Gln content was measured by a HPLC system. Effects of ASCT2 and/or GS inhibitor on tumor growth were investigated in xenograft models.

**Results:**

A significant heterogeneity of GC cells was observed with respect to their response to the treatment of ASCT2 inhibitor benzylserine (BenSer). Gln deprivation did not affect the BenSer-resistant cell growth due to endogenous GS expression, whose inhibition remarkably reduced cell proliferation. The differential in vitro sensitivity correlated with overall intracellular Gln content. Combined therapy with both ASCT2 and GS inhibitors produced a greater therapeutic efficacy than the treatment of either inhibitor alone. Furthermore, 77% human GC tissues were found to express moderate and high levels of ASCT2, 12% of which also co-expressed relatively high levels of GS.

**Conclusion:**

Gln mediates GC growth and the therapeutic efficacy of Gln-targeted treatment relies on distinct ASCT2 and GS expression pattern in specific gastric cancer groups.

## Introduction

Cancer cells have long been known to experience great metabolism alterations. Unlike normal cells which rely on mitochondrial oxidative phosphorylation, most cancer cells instead use aerobic glycolysis for ATP generation even in the presence of abundant oxygen supply, a phenomenon known as “the Warburg effect” (Warburg 1956). Elucidation of the metabolic differences and underlying mechanisms between cancer and normal cells could not only advance our understanding of fundamental cancer cell biology but also provide an important rationale for design of new therapeutic strategies aimed at selectively eliminating cancer cells by targeting their unique metabolism (Levine and Puzio-Kuter 2010). Over the past 30 years, increasing evidence has shown that glutamine (Gln) is an important metabolic substrate and energy source for many tumor cells which require Gln for their continued growth and survival, exhibiting so called “glutamine addiction” (Dang 2010; Wise and Thompson 2010). However, the complex metabolic logic of the proliferating cancer cells’ addiction to Gln, which goes far beyond satisfaction of energetic and biosynthetic needs, has only recently come into focus.

The solute-linked carrier family A1 member 5 (SLC1A5) gene encodes a Na^+^-dependent neutral amino acid transporter, ASCT2 (Grewer and Grabsch 2004). ASCT2 is the major transporter responsible for Gln uptake into rapidly proliferating cells, including cancer cells (McGivan and Bungard 2007; Wise and Thompson 2010). Suppression of Gln uptake by ASCT2 pharmacological inhibitors or by shRNA-mediated knockdown of ASCT2 has been shown to successfully inhibit cancer cell growth and proliferation in a variety of tumor types including non-small cell lung cancer, prostate cancer, breast cancer, melanoma, and human head and neck squamous cell carcinoma (Cui et al. 2015; Gong et al. 2014; Hassanein et al. 2013, 2015; Lu et al. 2016; van Geldermalsen et al. 2016). More recently, anti-tumor efficacy of a novel anti-ASCT2 humanized monoclonal antibody, KM8094, has also been demonstrated in patient-derived xenograft (PDX) mouse models of gastric cancer (Kasai et al. 2017). Intriguingly, from this study, a correlation between anti-tumor efficacy and low antigen expression as well as low basal levels of glutamine uptake has been identified, suggesting that ASCT2 expression level could be a potential predictive biomarker for KM8094. In addition, metabolomics analysis revealed clear differences in intracellular energy status and redox status between responsive and non-responsive PDX models. The catabolism of Gln is mediated by two different subtypes of mitochondrial glutaminase (kidney or liver-type encoded by GLS or GLS2, respectively) to become glutamate (Glu), a versatile metabolic intermediate that connects with many distinct biological processes such as oncogenic signaling events (Curthoys and Watford 1995; Gao et al. 2009; Wang et al. 2010). In this regard, targeting glutaminase by a small molecule inhibitor has been demonstrated to inhibit oncogenic transformation (Wang et al. 2010). Tumors can utilize multiple sources of Gln including de novo synthesis of Gln from intracellular Glu through glutamine synthetase (GS, encoded by GLUL, glutamate–ammonia ligase) catalyzing the reverse reaction of the glutaminases to meet their requirements for Gln (Newsholme et al. 2003). While a positive correlation of GS activity with cell survival and proliferation has been observed in some types of cancers (Kung et al. 2011; Tardito et al. 2015; Yang et al. 2016), few studies have focused on GS as a potential target for Gln-based cancer therapy.

Gastric cancer (GC) represents the fourth most common malignant neoplasm and the second leading cause of cancer death (Yang 2006). Approximately 70% of GC cases occur in developing countries, especially in East Asia (Bott et al. 2015). Irrespective of the fact that most of GC patients present with an advanced disease at diagnosis, the key treatment issue is that GC has very limited sensitivity to current chemotherapy (Chen et al. 2016; Orditura et al. 2014). Effective systemic treatment for patients with GC is apparently an unmet need in medical oncology. Therefore, exploration of novel approaches to treat GC is urgently sought. Here, we proposed that both ASCT2 and GS that coordinately control Gln homeostasis may serve as potential new therapeutic targets for GC. We identified two subgroups of GC cells either sensitive or resistant to ASCT2 inhibitor BenSer and determined that their sensitivity to BenSer or Gln deprivation was largely dependent on the expression levels of GS and correlated with overall intracellular Gln content. In vivo studies revealed that combined therapy with ASCT2 and GS inhibitor was more efficacious against ASCT2- and GS-expressing GC xenograft tumor growth than the treatment of BenSer or MSO alone. We also found that ASCT2 and GS were differentially expressed in GC tissues with the majority of GS specimen expressing high-level ASCT2 but low GS. Thus, ASCT2 and GS might represent attractive companion biomarkers for selecting GC population most likely responding to Gln-targeted therapy and further validation of these potential targets for therapy targeting molecularly defined subtypes of GC is warranted.

## Materials and methods

### Cell culture and clinical samples

Six distinct differentiated human gastric cancer cell lines including HGC-27 (undifferentiated), NUGC-3 (poorly differentiated), MKN-45 (poorly differentiated), MGC-803 (poorly differentiated), AGS (moderately differentiated), and MKN-74 (moderately differentiated) were obtained from the Type Culture Collection of the Chinese Academy of Sciences (Shanghai, China). All cell lines were maintained in RPMI-1640 (Gibco BRL, Grand Island, NY, USA) supplemented with 10% fetal bovine serum (FBS) except AGS in Ham’s F12 medium (Cellgro, Manassas, VA, USA) and incubated at an atmosphere containing 5% CO_2_ at 37 °C. Human gastric cancer tissues and their paired adjacent normal mucosa tissues were collected from the First Affiliated Hospital of Fujian Medical University (Fuzhou, China) from 2008 to 2010. Samples were immediately stored in liquid nitrogen after surgical resection. All samples were collected with patients’ informed consent and the study was approved by the Institutional Review Board of the First Affiliated Hospital of Fujian Medical University.

### Measurements of intracellular glutamine levels

Cells were plated into 24 well plates and grown to nearly 80% confluence. The extracellular medium was discarded and cells were quickly washed twice with HBSS (126.25-mM NaCl, 2-mM CaCl_2_, 3.0-M KCl, 1.25-mM NaH_2_PO_4_, 10-mM glucose, and 25-mM HEPES pH 7.4). Then, cells were incubated in 500 µl lysis buffer (1-mM DTT, 1-mM EDTA, and 10-mM NaOH) for 20 min and the lysis solution was harvested for consequent Gln measurement. Gln concentration was measured by a HPLC system as previously reported and was calculated from the amount of intracellular glutamine normalized to total amount of protein and expressed as nmol/mg protein.

### RNA extraction and real-time quantitative PCR

Total RNA was extracted from cultured cells using Trizol reagent (Ambion, Carisbad, CA, USA) and RNA was reverse transcribed to cDNA using RT Reagent Kit (TaKaRa, Dalian, China). Then, real-time quantitative PCR of cDNA was prepared using SYBR Premix EX Taq kit (Takara, Shiga, Japan) and amplification was performed on Mx3000P QPCR system (Agilent Technology, Santa Clara, CA, USA). The respective forward and reverse primers were used to detect the relative expression levels of the target genes by the 2^−△△ct^ method. The relative amount of target mRNA was normalized to β-actin. All the primers were designed by BioSune Biotechnology (Shang Hai) Co., Ltd.

### Western blot analysis

Cells were lysed with Western and IP cell lysis buffer (Beyotime, Shanghai, China) containing PMSF (Amresco, Solon, Ohio, USA) on ice for 30 min, and centrifuged at 12,000*g* for 10 min at 4 °C to collect the supernatant. Cellular protein (40 µg per lane) was separated by 10% SDS-PAGE and transferred onto a 0.45-µM PVDF membrane (AmershamHybond, GE Healthcare, München, Germany). The membrane was blocked with 0.5% bovine serum album (Amresco, Solon, Ohio, USA) at room temperature for 2 h. Then, the membrane was incubated with rabbit anti-ASCT2 (1:1000; Abcam), rabbit anti-glutamine synthetase (1:1500; Abcam) and mouse anti-GAPDH (1:1500; Cell Signaling Technology) overnight at 4 °C. The membranes were washed three times with TBS-T (0.1% Tween-20) for 10 min each at room temperature, incubated in secondary antibody for 30 min at room temperature and detected using enhanced chemiluminescence substrate detection solution (Lulong biotech, Xiamen China).

### Cell proliferation assay

Cells were seeded into 96-well plate at a density of 2 × 10^3^ cells per well and cultured for 24 in Gln-supplemented or free medium. Cells were continuously exposed to ASCT2 competitive inhibitor benzylserine (BenSer) (Sigma-Aldrich, St Louis, MO, USA) and/or GS selective inhibitor l-methionine sulfoximine (MSO) (Sigma-Aldrich, St Louis, MO, USA). The proliferation of cells was evaluated by the Cell Counting Kit-8 (CCK-8, Dojindo, Kuma-moto, Japan). 10 µl CCK-8 reagent was added into each well and incubated for 4 h. The absorbance from each well was determined using a microplate reader at the wavelength of 450 nm (Bio-Tek, Winooski, VT, USA).

### Colony formation assay

6 × 10^2^ cells were grown in 60-mm plates containing complete growth medium and BenSer (10 mM) and/or L-MS (1 mM) for 14 days. For Gln-starvation experiments, the culture was replaced with Gln-free medium on day 7 and continued incubation for additional 7 days. Then, the colonies formed that contained 50 or more cells were counted after staining with crystal violet for 5 min.

### Immunohistochemistry

Clinical specimens were dealt with dehydration of gradient ethanol and paraffin embedded, and processed into tissue sections with 4- µM thick for both tumor and paired adjacent normal gastric mucosa tissues. The sections were dewaxed in xylene and rehydrated in graded alcohol. Antigen retrieval was performed by 0.01-mol/L citrate buffer (pH 6.0) for 2 min. Endogenous peroxidase activity was inhibited with 3% hydrogen peroxide for 10 min. Sections were blocked by 5% BSA for 30 min at room temperature, and then incubated with rabbit anti-ACST2 (1:100; Abcam) and rabbit anti-Glutamine Synthetase (1:100; Abcam) at 4 °C overnight. The experimental procedure was performed according to the manufacturer’s instruction of the polink-2 plus Polymer HRP Detection System (ZSGB-bio, Beijing, China). Staining results were assessed by two pathologists independently.

### Animal studies

All work performed with animals was in accordance with and approved by the Institutional Animal Care and Use Committee (IACUC) at the Fujian Medical University (Approval No. 2016-030). The in vivo antitumor efficacy of ASCT2 and GS inhibitors were assessed in 5–8-week-old male athymic BALB/c nude mice bearing HGC-27 tumor xenografts. 2 × 10^6^ HGC-27 cells in 0.2 mL of RPMI 1640 medium were injected subcutaneously into the left and right posterior flank regions of each mouse. After the tumors were palpable, mice were randomly divided into four groups and the tumor volume was determined by the formula volume = length × width^2^/2. When the average tumor size in a group reached 100 mm^3^, the mice were treated with a single dose of vehicle control, BenSer (50 µg/kg), MSO (5 µg/kg) or the combination by the i.p. route. Then, the tumor size was measured every week for 4 weeks and plotted as a function of time to generate the in vivo growth curves. All animals were euthanized when the calculated tumor volume reached 1000 mm^3^ in either of the four groups.

### Statistical analysis

Data are presented as mean ± SEM. All two-group comparisons used Student’s *t* test or paired *t* test and analyzed by IBM SPSS statistics version 19 for Windows (IBM Corp., USA). Figures were generated by GraphPad Prism 5 (GraphPad Software, Inc., USA). A two-tailed *P* value < 0.05 was defined to be statistically significant.

## Results

### Sensitivity of various gastric cancer cells to ASCT2 inhibitor

**Fig. 1:**
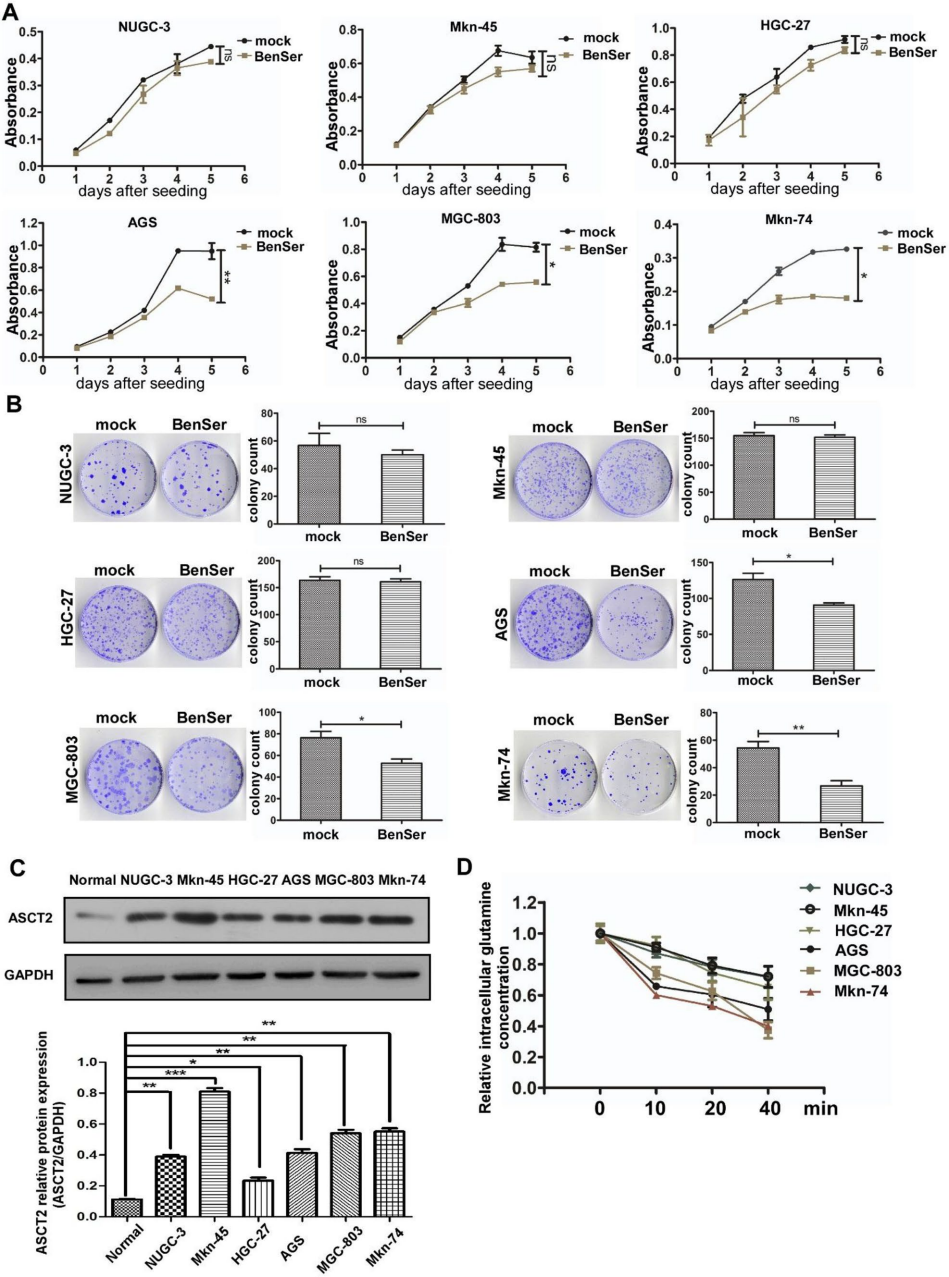
Gastric cancer cells show different responses to the ASCT2 inhibitor BenSer. **a** CCK-8 cell viability assay showing the effect of treatment with 10-mM BenSer on the growth of GC cell lines NUGC-3, Mkn-45, HGC-27, AGS, MGC-803, Mkn-74. **b** Colony formation assay showing showing the effect of treatment with 10-mM BenSer on the growth of the six GC cell lines. **c** ASCT2 protein expression in the six GC cell lines and normal gastric mucosa (Normal). GAPDH serves as a loading control. Bar graphs are the results of densitometric analysis of western blots showing the relative total ASCT2 expression. **d** Relative intracellular Gln levels in the six GC cell lines treated with 10-mM BenSer for the indicated time. Data were presented as mean ± SEM (*P < 0.05, **P < 0.01, ***P < 0.001, NS, *P* > 0.05)

Glutamine metabolism is essential for tumorigenesis and progression of various cancers through remodeling their glutamine metabolic pathways to fuel rapid proliferation. ASCT2 chemical inhibitors are well known for their capability of suppressing glutamine transport, cell growth and glutamine metabolism in vitro. We first set out to determine whether ASCT2 competitive inhibitor benzylserine (BenSer) could inhibit the growth of various gastric cancer cells. As shown in Fig. 1a, compared to the untreated controls exposure to 10 mM BenSer did not affect the proliferation of NUGC-3, Mkn-45, HGC-27 cells (designated hereafter as BenSer-resistant cells), whereas cell growth was significantly decreased in the BenSer-treated AGS, MGC-803, Mkn-74 cells (designated hereafter as BenSer-sensitive cells). To assess the long-term effects of BenSer on cell growth and survival, clonogenic assay was performed on the six GC cells. Likewise, treatment with BenSer reduced the clonogenic and reproductive potential of AGS, MGC-803, Mkn-74 cells but had no effect on colony formation of NUGC-3, Mkn-45, HGC-27 cells (Fig. 1b). We next determined whether ASCT2 expression was directly responsible for the observed sensitivity of the six GC cells to BenSer challenge. Intriguingly, while all the six GC cells expressed higher levels of ASCT2 protein as compared with normal gastric mucosa tissues, BenSer-induced cell inhibitory effects were not necessarily correlated with ASCT2 expression levels (Fig. 1c). Actually, the BenSer-resistant MKN-45 cells expressed the highest levels of ASCT2 among the six GC cells. To establish a causal link that might account for variable efficacy of BenSer in inhibiting cell growth, intracellular Gln levels were measured at various time points following BenSer exposure. As shown in Fig. 1d, the three BenSer-sensitive GC cells displayed lower intracellular Gln contents than the BenSer-resistant cells across all the time points examined. This result implicates that overall level of intracellular Gln is a major determinant of GC cell fate in response to ASCT2 inhibition.

### Effect of gln starvation on GC cell growth

**Fig. 2:**
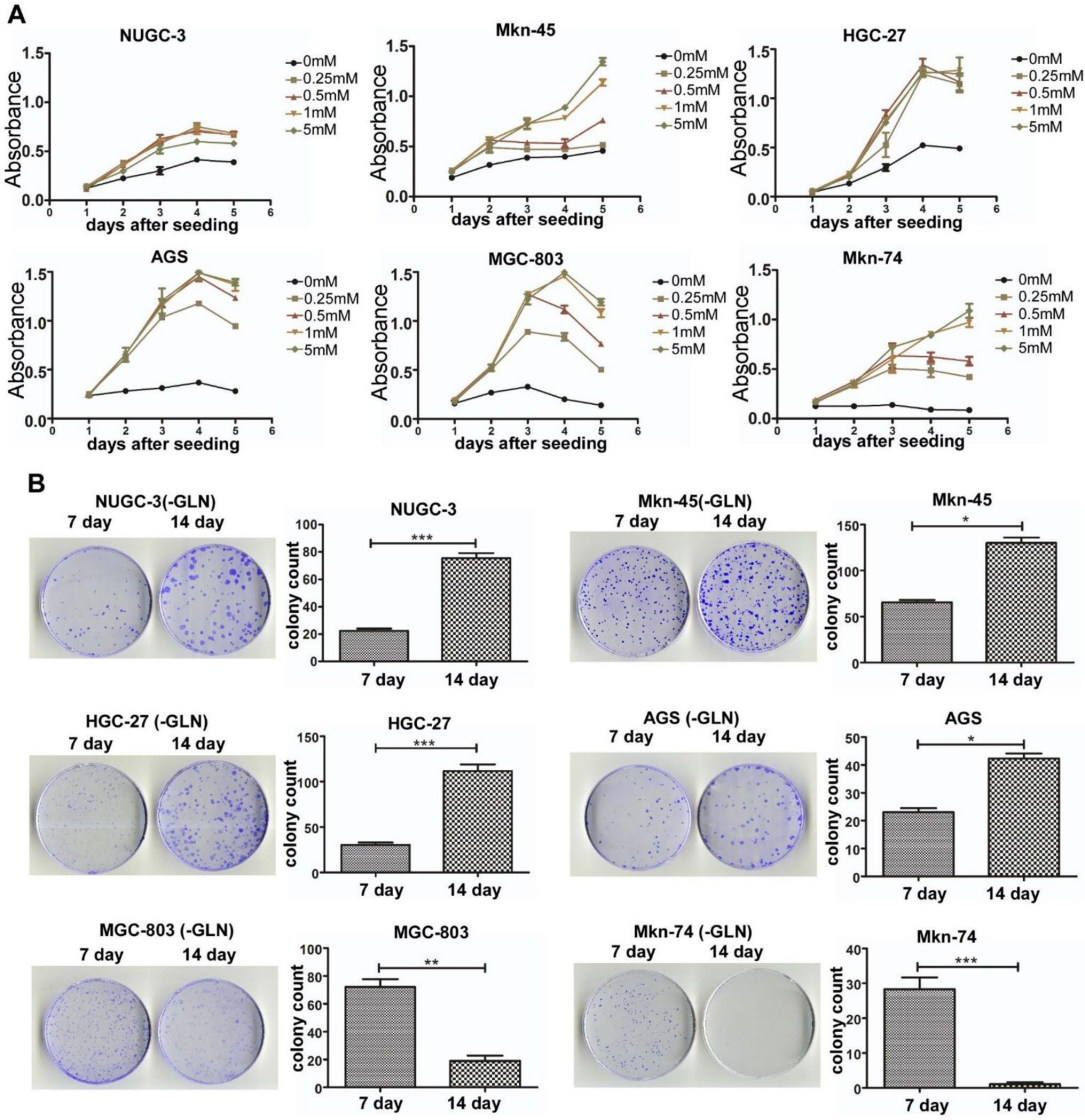
Effect of Gln withdrawal on the proliferation of BenSer-sensitive or -resistant GC cells. Dose–response curves determined by CCK-8 assay for the six GC cell lines incubated for 5 days in the medium with the indicated concentrations of Gln. **b** Clonogenic survival of the six GC cell lines under Gln starvation for 7 and 14 days. Data were presented as mean ± SEM (*P < 0.05, **P < 0.01, ***P < 0.001)

Since Gln is a versatile nutrient required for the survival and growth of a potentially large subset of tumors, we sought to determine whether its deprivation could differentially affect the growth of the BenSer-sensitive and -resistant GC cells. Cells were grown in media containing various concentrations of Gln for 5 days and the growth curves were determined by CCK-8 assay. As shown in Fig. 2a, Gln withdrawal reduced the proliferation of both BenSer-sensitive and -resistant GC cells in a Gln concentration dependent fashion. However, Gln depletion resulted in a complete growth inhibition on BenSer-sensitive cells, whereas deprivation of Gln only slowed down the proliferation rate of BenSer-resistant cells. Similarly, on the clonogenic cell survival assay (Fig. 2b) BenSer-resistant cells were able to retain their reproductive abilities to form more and larger colonies with time in the absence of exogenous Gln. In contrast, BenSer-sensitive cells were losing their proliferative and reproductive capacities upon Gln deprivation. These data indicated a differential reliance on exogenous Gln among the GC cells, whereby it appeared that only BenSer-sensitive cells required ASCT2-mediated glutamine uptake for cell growth and survival.

### Glutamine synthetase sustains BenSer-resistant GC cell growth under gln deprivation

**Fig. 3:**
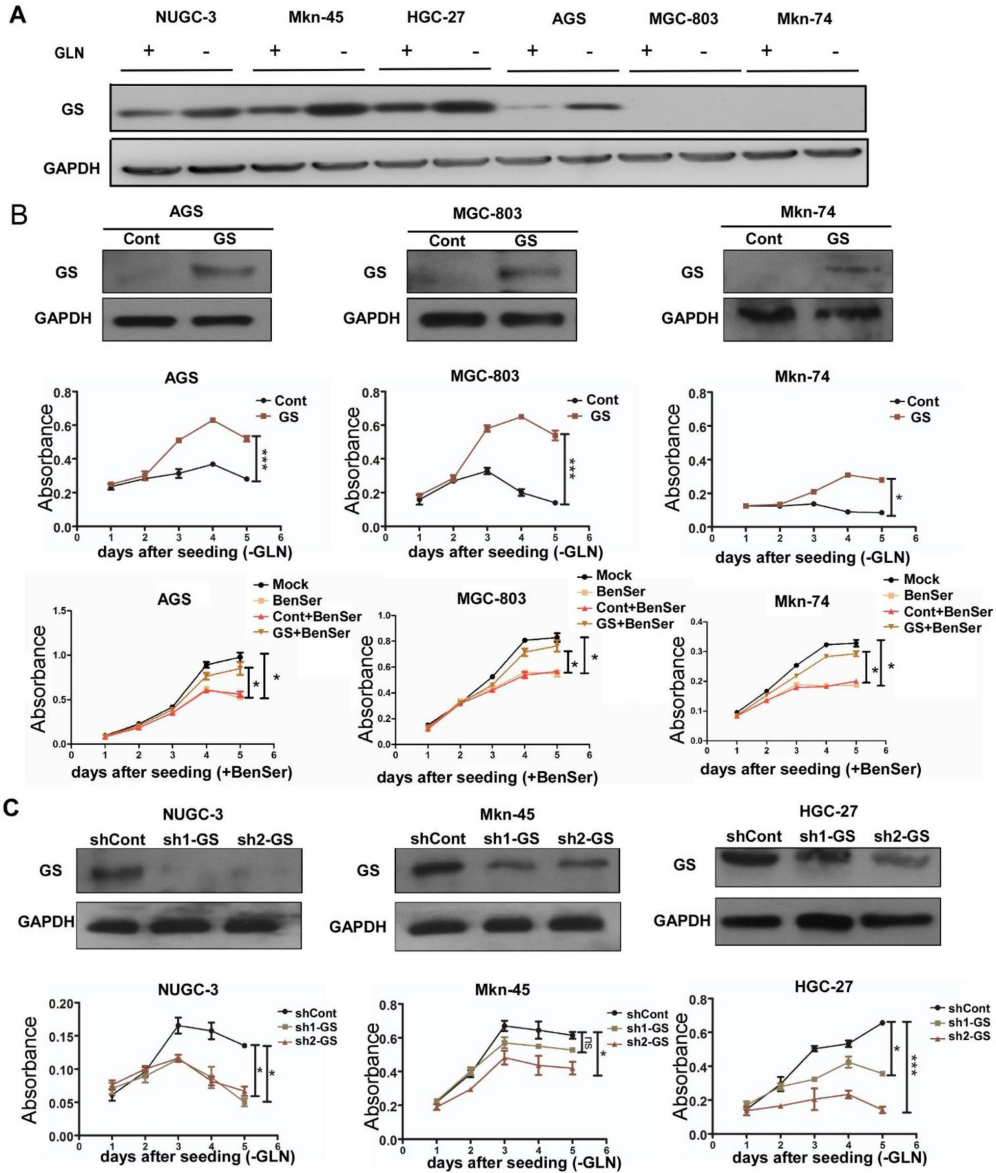
GS sustains cell growth during Gln starvation. **a** GS protein expression in the GC cells under Gln-deprived or -fed condition for 24 h. **b** Effect of overexpression of GS (upper row) in GS-deficient GC cells (AGS, MGC-803 and Mkn-74) on the cell proliferative ability upon Gln starvation (middle row), and on the sensitivity to BenSer in Gln-supplemented normal medium (bottom row). **c** Knockdown of GS in GS-proficient GC cells (HGC-27, Mkn-45 and NUGC-3) by two shRNA targeting GS reduced the cell proliferation under Gln starvation. Western blot analysis was performed to confirm stable overexpression or knockdown of GS in the GC cells. CCK-8 assay was used to assess the cell proliferative capacity. Data were presented as mean ± SEM (*P < 0.05, **P < 0.01, ***P < 0.001)

Given the observation that Gln deprivation did not affect the capacity of BenSer-resistant cells to divide and proliferate as evidenced by the clonogenic assay, we suspected that there must exist other mechanisms rather than ASCT2 contributing to regulate the growth of BenSer-resistant cells without a need of an exogenous Gln supply. Such an exogenous glutamine independence might result from increased expression of glutamine synthetase (GS) conferring the ability to synthesize glutamine from glutamate (Glu) endogenously. Therefore, we examined and compared GS protein expression across the six GC cells under Gln deprived or non-starved condition. As shown in Fig. 3a, Gln deprivation markedly increased GS expression levels in the BenSer-resistant cells but did not affect the expression of GS in the BenSer-sensitive cells. It is noteworthy that GS was not detectable in any of the three BenSer-sensitive cell lines, which may explain why the BenSer-sensitive cells were more dependent on extracellular Glu for growth. To corroborate a causal link between Gln biosynthesis and Gln-dependency, GS was overexpressed in the BenSer-sensitive cells which had displayed undetectable GS and high sensitivity to Gln deprivation. As expected, forced expression of GS in the BenSer-sensitive cells as confirmed by Western blot analysis dramatically restored proliferation of cells grown in glutamine free culture medium and these GS-overexpressing cells became resistant to BenSer in Gln-supplemented normal medium as compared to the empty vector-transfected cells (Fig. 3b). In line with this, knockdown of GS by either of two shRNA sequences targeting GS in the BenSer-resistant cells significantly lowered cell proliferation upon Gln withdrawal (Fig. 3c). These results imply that under Gln deprivation GS expression is essential for GC cells to sustain their growth advantage.

### Potentiation of in vitro and in vivo antitumor activity by combination treatment with BenSer and MSO

**Fig. 4:**
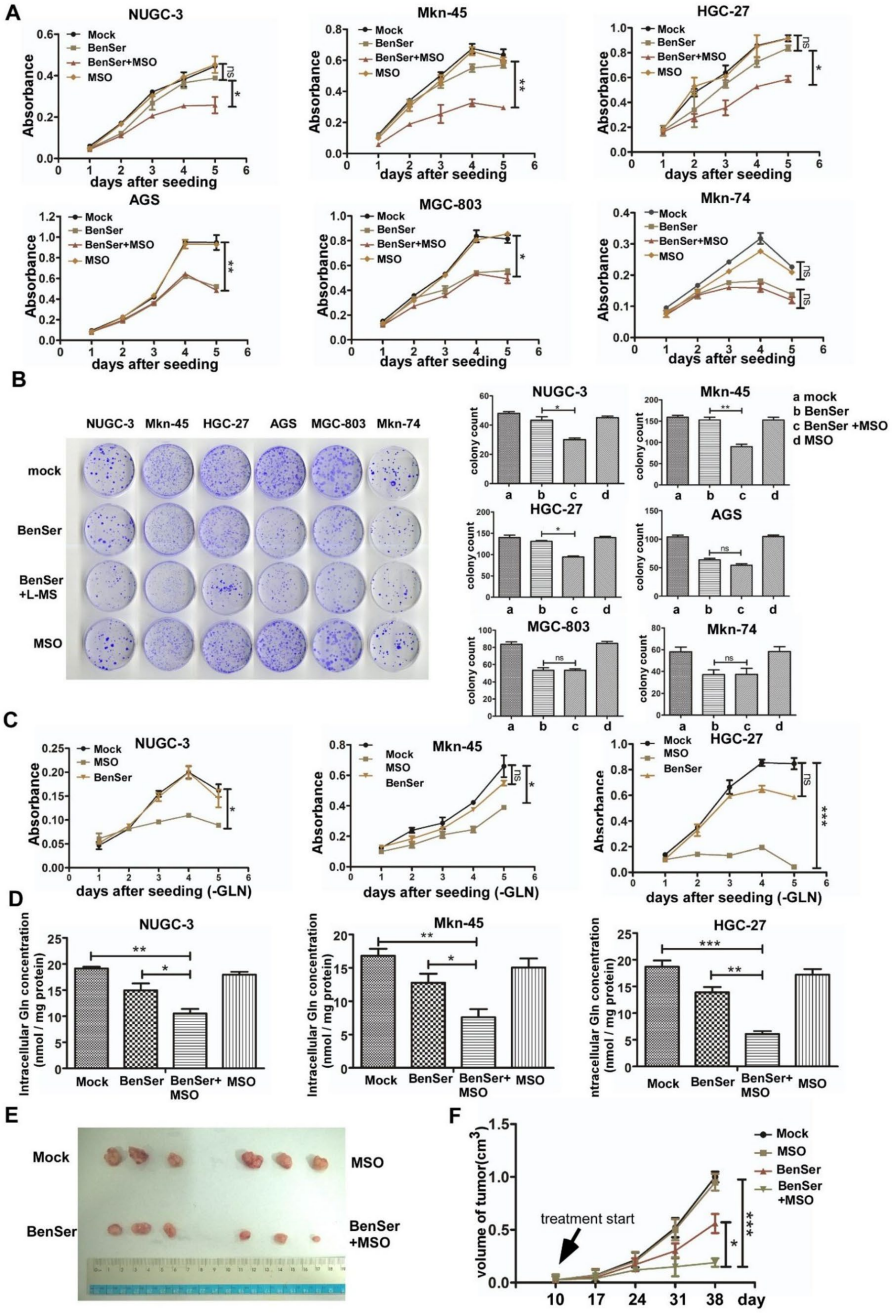
Combination treatment of BenSer with MSO has a greater therapeutic effect on ACST2- and GS-expressing GC cells in vitro and in vivo. **a** Growth curves of the six GC cells treated with 10-mM BenSer or 1-mM MSO alone or the combination. **b** Clonogenic and reproductive potential of the six GC cells treated with 10-mM BenSer or 1-mM MSO alone or the combination. **c** Proliferation of Gln-starved GS-expressing GC cells treated with 10-mM BenSer or 1-mM MSO. **d** Intracellular Glu levels in the GS-expressing GC cells treated with 10-mM BenSer or 1-mM MSO alone or the combination. **e** Photograph of the xenografts removed from nude mice i.p. injected with a bolus dose of 50-µg/kg BenSer, 5-µg/kg MSO or the combination at 38 days after tumor implantation. **f** Growth of tumors in the four experimental groups. Data were presented as mean ± SEM (*P < 0.05, **P < 0.01, ***P < 0.001, NS, *P* > 0.05)

Based on the distinct expression patterns of ASCT2 and GS in the six GC cells, we went further to test the therapeutic strategy and efficacy of inhibiting both ASCT2 and GS by BenSer and l-methionine sulfoximine (MSO), a selective irreversible inhibitor of GS, in the GC cells grown in Gln-supplemented normal media. As shown in Fig. 4a, continuous exposure of BenSer-resistant cells to BenSer or MSO alone barely affected cell proliferation as compared to the untreated controls. However, combined treatment with BenSer and MSO significantly hindered proliferation. As for BenSer-sensitive cells, while MSO did not change the growth rate of cells treatment with BenSer produced a significant reduction in cell proliferation and the combination of BenSer with MSO achieved no greater inhibition. Similar results were obtained with colony formation assay (Fig. 4b). To verify that GS was indeed indispensable for Gln biosynthesis to sustain the growth of Gln-starved cells, BenSer-resistant cells cultured in Gln-free medium were incubated with BenSer or MSO and growth curves measured over 5-day period. As anticipated, in the absence of exogenous Gln, inhibition of glutamine transporter ASCT2 had no effect on cell growth. Conversely, GS inhibitor MSO remarkably reduced cell proliferation (Fig. 4c). To determine whether the differences in the growth-inhibitory effects of BenSer and MSO were attributable to different intracellular Gln levels, the amount of intracellular Gln was measured in BenSer-resistant cells after exposure to either BenSer or MSO alone or the combination. As shown in Fig. 4d, treatment with BenSer or MSO alone caused only moderate decrease in Gln levels, whereas the combined therapy resulted in a marked drop in intracellular Glu. These results clearly indicate that intracellular Gln reduction due to both impaired Gln uptake and biosynthesis is a key determinant for GC cell growth inhibition probably by limiting the access of Gln to key signaling targets capable of triggering and sustaining proliferation.

To determine whether the difference in growth-inhibitory effects of BenSer and/or MSO measured in vitro translated into a difference in tumor responsiveness in vivo, the NUGC-3 cells were xenografted subcutaneously into BALB/c nu/nu mice. The resulting tumors were allowed to grow to an average volume of 100 mm^3^, and the mice were then treated with either BenSer or MSO alone or the combination of the two drugs by a single i.p. injection. Figure 4e shows that MSO had no antitumor activity while BenSer produced a moderate response in the NUGC-3 tumors. However, the combined therapy at the equitoxic doses of BenSer and MSO significantly inhibited tumor growth as compared with BenSer treatment alone. Therefore, consistent with the effect of BerSer and MSO on the growth of NUGC-3 cells in vitro, co-targeting ASCT2 and GS produced a greater therapeutic efficacy in vivo as well.

### Expression of ASCT2 and GS in gastric cancer tissues

**Fig. 5:**
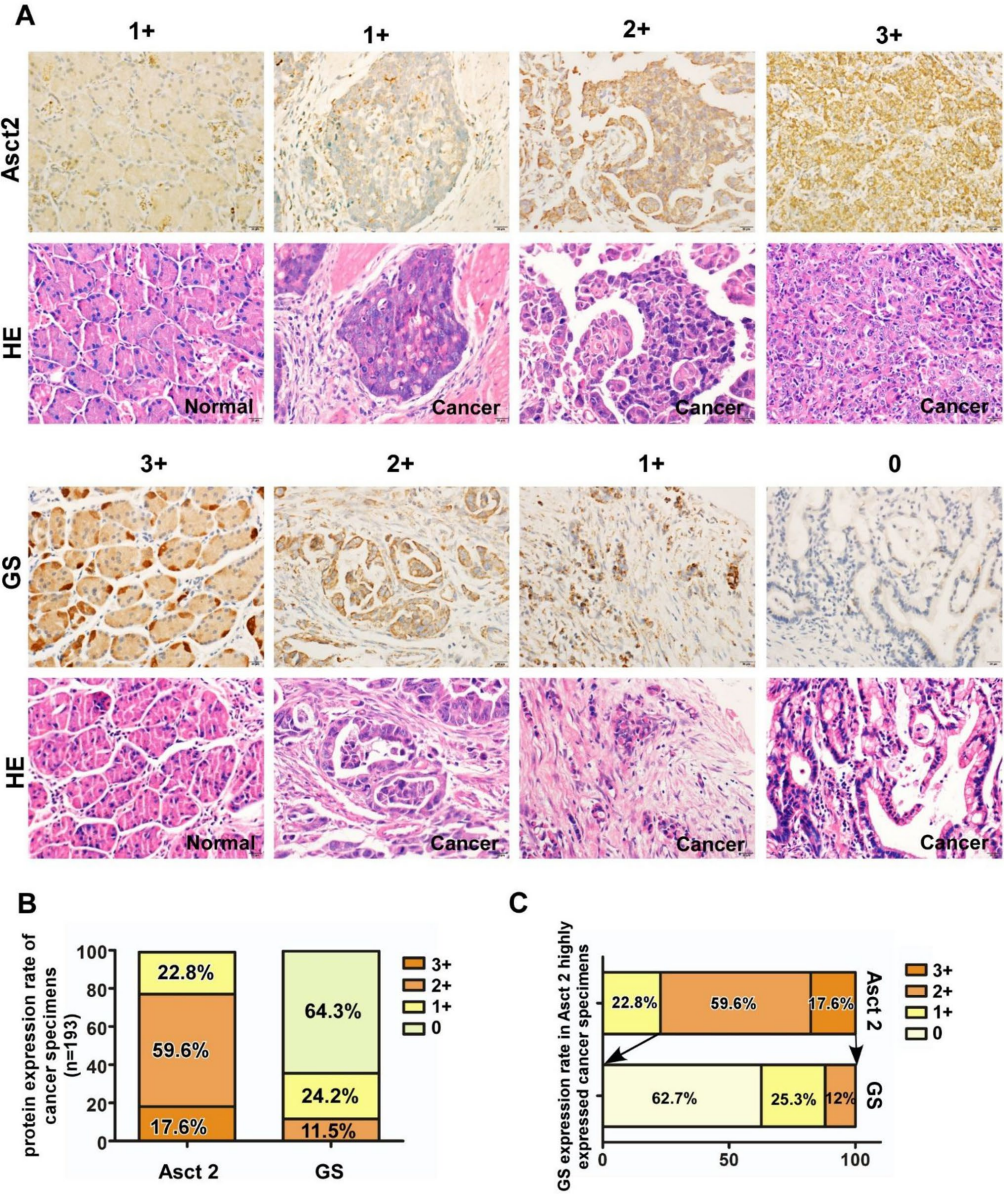
ASCT2 and GS protein expression in tumors from GC patients. **a** Representative images of IHC staining for ASCT2 and GS in 193 pairs of primary GC specimen and adjacent normal mucosa tissues. The scale of 0, 1+, 2+ and 3+ staining on ASCT2 and GS in GC tissue sections were shown. **b** ASCT2 and GS expression rate in GC tissues according to the staining score ranging from 0+ to 3+. **c** GS expression rate in ASCT2 highly expressed GC specimens

Finally, we analyzed the expression of ASCT2 and GS in normal gastric tissues and GC specimens by immunohistochemistry. As shown in Fig. 5a, ASCT2 was significantly overexpressed in the GC samples compared with adjacent non-cancerous gastric mucosa. In contrast, a significantly higher level of GS expression was observed in normal tissues than in tumor tissues. Based on the extent of staining in 193 GC tumor samples, there was 22.8% (*n* = 44/193), 59.6% (*n* = 115/193) and 17.6% (34/193) of the GC samples, respectively, that expressed low, moderate and high level of ASCT2. However, GS expression was not, or very weakly detected in the majority (86.5%, *n* = 167/193) of the GC samples (Fig. 5b). Of note, in the tumor tissues expressing moderate and high levels of ASCT2 (*n* = 149), 12% (*n* = 18) of which also co-expressed relatively high levels of GS.

## Discussion

Cancer cells have been well known for profound alterations in their metabolism, as exemplified by the Warburg effect, a phenomenon of cancer cells with elevated aerobic glycolysis (Kroemer and Pouyssegur 2008; Vander Heiden et al. 2009). Reprogramming of the cellular energy metabolism constitutes an emerging hallmark of cancer (Pavlova and Thompson 2016). Glutamine is a key metabolic substrate in cancer cells and is critical for tumor development, progression, and response to therapy (Lukey et al. 2013). Thus, elucidation of Gln metabolic differences would not only advance our understanding of fundamental cancer cell biology but also help characterize specific tumor groups to allow design of personalized therapies.

Gastric cancer is an aggressive tumor with low response to current standard of care cytostatic anticancer drugs. Therefore, modulation of the aberrant glutamine metabolism, if any, in GC may be a useful strategy for GC therapies. Since increased glutamine transporters account for glutamine addiction in most cancer cells we first attempted to test the sensitivity of six different gastric cancer cell lines to BenSer, a specific glutamine transport inhibitor. Interestingly, the sensitivity profile seemed to allow us to distinguish and possibly stratify the GC cells into two groups, one responsive to BenSer treatment and the other poorly responsive to the inhibitor. Unexpectedly, the sensitivity was not necessarily associated with ASCT2 expression levels but did correlate with intracellular Gln levels. This observation raised the question of whether glutamine dependence of GC cells for growth was also mediated by the cell-type-specific expression of glutamine synthetase (GS). To this end, the six GC cells were grown in Gln-free medium to deplete extracellular Gln supply, then the expression of GS and cell viability were determined in BenSer-sensitive and -resistant GC cells. We found that GS was barely expressed in BenSer-sensitive cells but abundant in BenSer-resistant cells, and that Gln deprivation led to the complete proliferative block in BenSer-sensitive cells without affecting the growth of BenSer-resistant cells. This finding suggests that GS was essential for the adaptation to the metabolic stress caused by Gln starvation and this adaptive importance of GS was consistent with the observation that exogenous Gln-deprived BenSer-resistant cells displayed an increased level of GS. The essential role of GS in determining cell viability under Gln deprivation was further supported by the fact that knockdown of GS in BenSer-resistant cells diminished cell proliferation, whereas forced expression of GS in BenSer-sensitive cells promoted growth.

A further question we asked was whether co-targeting of both ASCT2 and GS in the GC cells could produce a better therapeutic effect both in vitro and in vivo. On the availability of exogenous Gln that can be transported into the cell, GS-expressing BenSer-resistant cells were more sensitive to the combined treatment of ASCT2 and GS than used alone, indicating that both uptake of exogenous Gln and generation of endogenous Gln were limited as reflected by the significantly decreased levels of Gln inside the cells. However, for BenSer-sensitive cells with undetectable GS, GS inhibition by MSO did not further enhance the anti-proliferative of ASCT1 since the cells did not reply on endogenous synthesis of Gln for growth. Of important note, under Gln-deprived condition where BenSer-resistant cells fully depended on glutamine synthesized intracellularly by GS, MSO dramatically impaired cell proliferation. Furthermore, mouse xenograft model was employed to examine whether MSO and BenSer combination treatment could produce a higher efficacy against both ASCT2 and MS-expressing tumors. While BenSer produced a partial inhibition on tumor growth and MSO showed no treatment benefit, BenSer and MSO co-treatment significantly suppressed tumor growth. Collectively, these data support the possibility that such combination therapy may be an effective approach for treatment of GC patient population whose tumors express both ASCT2 and GS.

However, it should also be recognized that as energy metabolism and its regulatory machinery are evolutionarily conserved and shared by various normal cells, metabolism-related inhibitors such as BenSer and MSO may have a high probability of affecting the normal cells to produce adverse effects as well. In the history of targeting glutamine addiction, a number of pharmacologic agents that show promising results in preclinical studies have failed in clinical trials due to dose-limiting toxicities such as neurotoxicity, gastrointestinal toxicity, and myelosuppression (Ahluwalia et al. 1990). Therefore, whether the metabolic alterations in cancer cells can be targeted efficiently and safely in the clinic remains an unsolved issue. A more comprehensive understanding of the metabolic differences between cancer and normal cells through detail investigation might pave the way to achieve this goal. Furthermore, a combination of conventional chemotherapeutic agents and metabolic modulators may enhance therapeutic activity and should be further evaluated.

Immunohistochemistry of primary human GC samples demonstrated that ASCT2 was expressed to various extent in GC tissues of all 193 cases examined, which was in sharp contrast to adjacent normal gastric mucosa tissues negative for ASCT2 staining. ASCT2 is frequently upregulated in multiple cancers and inhibition of ASCT2-mediated glutamine uptake by pharmacological inhibitors of ASCT2 or by shRNA-mediated knockdown of ASCT2 has been shown to successfully inhibit cancer cell growth and proliferation (Cui et al. 2015; Gong et al. 2014; Hassanein et al. 2013, 2015; Lu et al. 2016; van Geldermalsen et al. 2016). Surprisingly, normal gastric tissues expressed a significantly higher level of GS than tumor tissues. Previous studies showed that multiple oncogenic signaling pathways have been found to increase the expression of GS and positive correlation of GS activity with cell survival and proliferation have been also observed in Myc-driven cancers (Bott et al. 2015; van der Vos et al. 2012; Yang et al. 2016). While ASCT2 immunohistochemical staining may be a useful pathological marker for GC as well, the biological significance of GS expression in gastric carcinogenesis and progression remains to be elucidated. It may be noteworthy that a correlation between ASCT2/GS expression and clinical outcome is not available in TCGA database. To investigate their prognostic significance, a large cohort including different types and stages of gastric cancers and associated with clinical data would be required. Regardless, from therapeutic point of view, selection of GC patients on the basis of ASCT2 and GS expression profile may prove to be a beneficial approach to target tumors for achieving desirable therapeutic outcomes.

In summary, this report demonstrates that Gln is a key driver of GC growth and Gln-targeting therapeutics may be of special clinical utility for gastric cancers with few current therapeutic options. Moreover, stratification of GC tumors based on ASCT2 and GS expression is important for personalized therapeutic strategies against the treated tumors by blocking glutamine transporters and/or inhibiting GS activity.
